# Different equations for estimating age-related changes of glomerular filtration rate in the healthy population

**DOI:** 10.1186/s12882-023-03397-7

**Published:** 2023-11-17

**Authors:** Lu Wei, Xue Shen, Juan Zhang, Zhenzhu Yong, Qun Zhang, Weihong Zhao

**Affiliations:** 1 Division of Nephrology, Department of Geriatrics, The First Affiliated Hospital of Nanjing Medical University, No. 300 Guangzhou Road, Nanjing, 210029 Jiangsu China; 2 Department of Health Management, The First Affiliated Hospital of Nanjing Medical University, Nanjing, Jiangsu China

**Keywords:** Healthy, Aging, Glomerular filtration rate, Equation

## Abstract

**Background:**

Identifying age-related trend of estimated glomerular filtration rate (eGFR) is necessary to assess whether kidney function is healthily aging. This study aimed to investigate the application of CKD-EPI, FAS, and Xiangya equations for the aging estimation of eGFR in the healthy Chinese individuals.

**Methods:**

A total of 36,911 healthy individuals were enrolled in this study. We grouped every ten years to observe the trend of eGFR with aging and investigated decline rate of it by general linear regression analysis in each age-groups. Agreement between equations was determined by intraclass correlation coefficient (ICC) and Bland–Altman plot. We calculated reference interval in each age-group. We further analyzed above statistical indicators in males and females.

**Results:**

The eGFR by CKD-EPI, and Xiangya equation started to decline from the age of 18. Whereas eGFR by FAS equation remained stable under 40 years, then decreased more rapidly. Compared with males, the females had a higher level but a faster decline rate of eGFR with aging. Agreement analysis revealed good agreement between CKD-EPI and FAS equations (ICC 0.818–0.920). Agreement between Xiangya and CKD-EPI or FAS equations was poor to moderate in most of the population under 70 years old (ICC 0.282–0.786), but good in individuals above 70 years (ICC 0.769–0.881).

**Conclusions:**

The trend of eGFR with aging was different by CKD-EPI, FAS, and Xiangya equations in the healthy Chinese. It may be necessary to take these equations- or age-related differences into consideration when assessing kidney function in primary health care and clinical practice.

**Supplementary Information:**

The online version contains supplementary material available at 10.1186/s12882-023-03397-7.

## Introduction

Aging population is one of the major challenges worldwide [1]. The kidney is one of the organs prone to aging, and a gradual decline in kidney function occurs with age [2]. Glomerular filtration rate (GFR) is an important indicator of kidney function. Calculating decline rate of GFR is necessary to determine whether it exceeds the physiological rate of decline. Therefore, it is important to assess this physiological rate of decline and the reference intervals for normal GFR at all ages both in primary medical care and in clinical practice.

Previous studies, using exogenous filtration markers or clearance techniques to measure GFR (mGFR), have investigated a declining trend with aging based on healthy kidney donors [3–5]. These methods, while accurate, are invasive, expensive, and cumbersome to operate. They could not be widely implied in the healthy screening and primary medical care. To estimate GFR more easily, several equations were developed and applied to estimate GFR [6–9].

The chronic kidney disease epidemiology collaboration (CKD-EPI) equation based on serum creatinine (SCr) was developed in 2009 and recommended by the 2012 Kidney Disease Improving Global Outcomes (KDIGO) guideline for assessing GFR [10]. Due to few samples of Asians and a certain number of patients with chronic kidney disease in the development dataset, its accuracy in estimating GFR is not ideal in the Chinese, especially in healthy population [8, 11]. In 2016, Pottel et al. developed a full-age-spectrum (FAS) equation recruiting 6,870 healthy European subjects, which had continuity throughout the age spectrum and avoided conversion for estimation equations between different age groups [6]. The FAS equation performed fairly in our previous studies [12, 13]. Considering the influence of race on the accuracy of the equations, Xiangya equation was developed based on a multi-ethnic Chinese population in 2019 [7, 14]. Due to the lack of substantial evidence and guideline recommendations, the FAS and Xiangya equations are not widely used in clinical practice. The values returned by these equations were different and not comparable. To date, few study focused on the age-related trend of estimated GFR (eGFR) by above equations in the healthy population. Further exploration of these equations could provide certain reference for clinical practice in the evaluation of kidney function.

Thus, the present study was to investigate the application of CKD-EPI, FAS, and Xiangya equations in assessing age-related changes of eGFR levels in the healthy Chinese, and to preliminarily assess the consistency of these equations in estimating GFR. Doctors will be provided with the range and rate of kidney function decline for each age group based on each formula.

## Material and methods

### Study design and participants

This study is a retrospective cross-sectional study on Chinese adults (age≧18 years) at the Health Management Center of The First Affiliated Hospital of Nanjing Medical University from January 2018 to January 2020. This study was approved by the ethics committee of the First Affiliated Hospital of Nanjing Medical University and approved with a waiver of informed consent as the research involves no more than minimal tangible or intangible risk to the subjects (No. 2018-SR-181).

The inclusion criteria were: (1) BMI:18.5-30 kg/m^2^, (2) No history of hypertension or blood pressure (BP) < 140/90 mmHg, (3) No history of diabetes or fasting blood glucose (FBG) < 7.0 mmol/L, (4) No history of kidney disease, absence of proteinuria and hematuria, SCr ≤ 133.0μmol/L, (5) No history of the cardio-cerebrovascular disease, malignant tumor, thyroid disorders, and systemic disease, and (6) Serum uric acid (UA), alanine aminotransferase (ALT), low-density lipoprotein cholesterol (LDL-C), high-density lipoprotein cholesterol (HDL-C), and total cholesterol (TC) were in reference range according to guidelines for the Chinese. The exclusion criteria were absence of SCr value and being pregnant.

### Variables and measurement

Basic demographic information (gender, age, and past medical history) was collected using a physician-administered questionnaire by nurses and staff physicians. After the subject sat quietly for 5 min at least, nurses used a sphygmomanometer to measure the blood pressure of the left brachial artery one times. Body mass index (BMI) was calculated as measured weight (kg) divided by measured height (m) squared.

Blood samples were collected in the morning after an overnight fast of at least 8 h. All the fasting blood samples were assayed on Beckman AU5800 automatic bio-chemical analyzer (Beckman Co., Ltd., USA), in strict accordance with the instructions of the apparatus. SCr was measured using the enzymatic method (sarcosine oxidase-PAP, Shanghai Kehua Bio-engineering Co., Ltd., China) with a reference range of 0.5–1.5 mg/dL, which is traceable to National Institute Standardized Technology creatinine standard reference material 909b. The intra- and inter-assay coefficients of variations for creatinine assay were consistently ≦6%, with stable quality control. All enzyme activities were measured at 37 °C.

### Estimation of GFR

eGFR was evaluated by the following equations: (1) eGFR_CKD-EPI_: eGFR = 141 × (SCr/0.9)^−0.411^ × 0.993^age^ (males and SCr ≤ 0.9 mg/dL), eGFR = 141 × (SCr/0.9)^−1.209^ × 0.993^age^ (males and SCr > 0.9 mg/dL), eGFR = 144 × (SCr/0.7)^−0.329^ × 0.993^age^ (females and SCr ≤ 0.7 mg/dL), eGFR = 144 × (SCr/0.7)^−1.209^ × 0.993^age^(females and SCr > 0.7 mg/dL). (2) eGFR_FAS_: eGFR = 107.3/(SCr/Q_SCr_) (for 2 ≤ age ≤ 40 years), eGFR = 107.3/(SCr/Q_SCr_) × 0.988^(age−40)^ (for age > 40 years) (female: Q_SCr_ = 0.70 mg/dl; male: Q_SCr_ = 0.90 mg/dl). (3) eGFR_Xiangya_: eGFR = 2374.78 × SCr^−0.54753^ × age^−0.25011^ × (0.8526126 if female). SCr was written in mg/dL when calculating eGFR using CKD-EPI and FAS formulas but was converted to µmol/l using Xiangya equation.

### Statistical analysis

All participants were stratified into six age groups: 18–29, 30–39, 40–49, 50–59, 60–69, and ≥ 70 years old. Continuous variables were represented as mean (standard deviation (SD)), and categorical variables were represented as proportion. T-test and Welch’s ANOVA test were adopted to compare the differences of continuous variables. General linear regression analysis was applied to calculate the annual eGFR decline rate. We plotted the scatter diagrams to directly reflect the different values in subgroups according to SCr.

Agreement between these equations was analyzed by Bland–Altman diagrams and intraclass correlation coefficient (ICC). Bland–Altman plots display for individual the difference between two of the three equations against their mean. The mean difference was used as bias to compare the equations with each other. 95% Limits of Agreement (LoA) between the equations were defined as mean difference ± 1.96 SD of the differences and were used as a measure of the variability of the bias. These values represented the range within which 95% of the differences were included. ICC values of < 0.5, 0.5–0.75, 0.75–0.9, and > 0.90 indicate poor, moderate, good, and excellent agreement, respectively. Considering the slight differences in the equations between males and females, we further conducted the above statistical analysis in subgroups according to gender. Finally, we calculated the reference intervals using mean ± 1.96 SD in age-gender subgroups. Two-tailed *P* < 0.05 was considered statistically significant.

Data were missing for BMI (5846, 5.62%), blood pressure (5581, 5.37%), FBG (2935, 2.82%), and UA (172, 0.17%). Missing values were imputed by EM algorithm. Statistical analyses were performed using the SPSS software version 25.0 (IBM Inc., USA) and MedCalc for Windows (version 19.6.1.0; MedCalc Software, Mariekerke, Belgium). The graphic drawing was implemented in Graphpad Prism 8.0.2 (GraphPad Software Inc., San Diego, CA, USA).

## Results

**Fig. 1:**
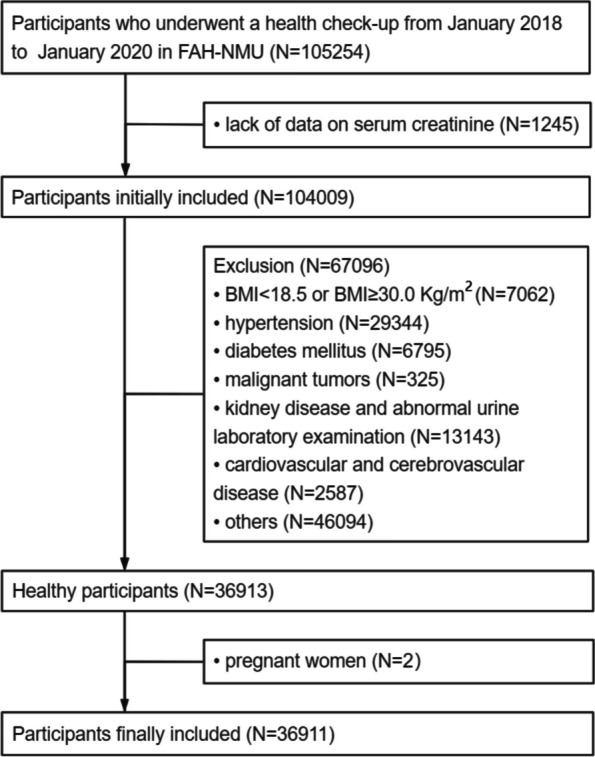
The flowchart for the eligible participates. *FAH-NMU:* the First Affiliated Hospital of Nanjing Medical University, *BMI:* body mass index

**Fig. 2:**
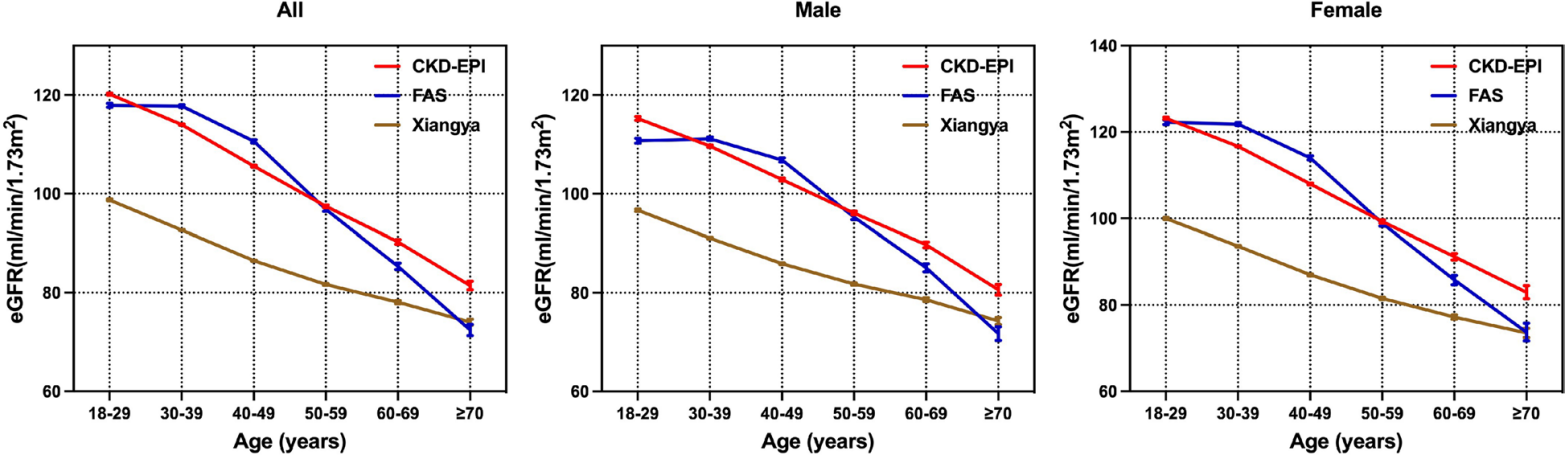
Trend of eGFR by CKD-EPI, FAS, and Xiangya equations with aging in healthy population. eGFR level was presented as mean and 95% confidence interval in each age-group. eGFR: estimated glomerular filtration rate. *CKD-EPI*: chronic kidney disease epidemiology collaboration equation based on serum creatinine; *FAS:* full age spectrum based on serum creatinine and age

A total of 36,911 healthy people, who met the eligibility criteria, were enrolled in this study. The flowchart diagram was shown in Fig. 1. The mean age of the total population was 38.9 years. Blood pressure and metabolic indicators vary with aging. The mean of SCr was 0.74 mg/dL (SD: 0.15) in the total population. BUN and SCr, as markers of kidney function, gradually increase with aging in subjects older than 40 years old. Characteristics of subjects in age groups were shown in Table 1, and characteristics of subgroups according to gender were shown in Supplementary Table 1. eGFR calculated by CKD-EPI level was relatively higher in groups aged 18–29 years and 50 years and over, compared to the other two equations. eGFR by FAS equation level was higher in groups aged 30–49 years (Fig. 2).

**Table 1:** Baseline characteristics of included participants

Variable	All subjects	18-29 years	30-39 years	40-49 years	50-59 years	60-69 years	≥ 70 years
	(*n* = 36911)	(*n* = 7849)	(*n* = 14377)	(*n* = 8328)	(*n* = 4342)	(*n* = 1525)	(*n* = 490)
male [n (%)]	16096 (43.61%)	2955 (37.65%)	5474 (38.07%)	3930 (47.19%)	2480 (57.12%)	943 (61.84%)	314 (64.08%)
SBP (mmHg)	116.80 (11.31)	116.03 (11.01)	114.92 (11.00)^*^	117.05 (11.25)^*^	120.25 (10.88)^*^	123.81 (10.79)^*^	127.66 (9.47)^*^
DBP (mmHg)	71.79 (8.17)	70.78 (7.83)	70.94 (7.98)	72.52 (8.39)^*^	74.24 (8.26)^*^	74.28 (7.91)	70.97 (7.99)^*^
BMI (kg/m^2^)	22.74 (2.39)	22.08 (2.34)	22.57 (2.38)^*^	23.10 (2.32)^*^	23.37 (2.27)^*^	23.55 (2.33)	23.61 (2.45)
HDL (mmol/L)	1.44 (0.27)	1.44 (0.26)	1.43 (0.26)	1.45 (0.28)^*^	1.43 (0.28)	1.43 (0.27)	1.42 (0.28)
LDL (mmol/L)	2.95 (0.53)	2.82 (0.52)	2.91 (0.52)^*^	3.03 (0.50)^*^	3.12 (0.50)^*^	3.07 (0.55)^*^	2.89 (0.64)^*^
TG (mmol/L)	1.08 (0.41)	0.96 (0.37)	1.04 (0.40)^*^	1.13 (0.42)^*^	1.20 (0.41)^*^	1.26 (0.43)^*^	1.20 (0.39)^*^
TC (mmol/L)	4.88 (0.69)	4.69 (0.70)	4.82 (0.69)^*^	5.00 (0.66)^*^	5.11 (0.64)^*^	5.07 (0.71)	4.84 (0.83)^*^
FBG (mmol/L)	5.07 (0.45)	4.93 (0.40)	5.02 (0.41)^*^	5.12 (0.44)^*^	5.25 (0.51)^*^	5.39 (0.53)^*^	5.55 (0.59)^*^
ALB (g/L)	45.43 (2.27)	46.39 (2.18)	45.70 (2.14)^*^	44.87 (2.21)^*^	44.62 (2.10)^*^	44.09 (1.99)^*^	42.87 (2.26)^*^
UA (umol/L)	298.69 (61.96)	300.17 (62.03)	293.96 (62.40)^*^	295.71 (62.82)	310.47 (57.76)^*^	313.22 (57.77)	314.64 (57.92)
BUN (mmol/L)	4.75 (1.12)	4.53 (1.09)	4.64 (1.07)^*^	4.81 (1.11)^*^	5.15 (1.13)^*^	5.25 (1.14)^*^	5.41 (1.30)
SCr (mg/dL)	0.74 (0.15)	0.72 (0.16)	0.72 (0.15)	0.75 (0.15)^*^	0.78 (0.15)^*^	0.79 (0.15)^*^	0.82 (0.16)

Values were presented as mean (standard deviation) or percent. *SBP* Systolic blood pressure, *DBP* Diastolic blood pressure, *BMI* Body mass index, *HDL-C* High density lipoprotein cholesterol, *LDL-C* Low-density lipoprotein cholesterol, *TG* Triglyceride, *TC* Total cholesterol, *FBG* Fasting blood glucose, *ALB* serum albumin, *UA* Serum uric acid, *BUN* Blood urea nitrogen

^*^P < 0.05, compared with former age spectrum

eGFR yielded a declined trend with aging, but the nodes and rates of decline were different (Fig. 2). eGFR_CKD-EPI_ gradually declined from 120.2 mL/min/1.73 m^2^ in subjects aged 18–29 years to 81.5 mL/min/1.73 m^2^ in individuals older than 70 years old, with decline rate of 0.82 mL/min/1.73 m^2^/year. eGFR_Xiangya_ gradually decrease from 98.7 to 74.0 mL/min/1.73 m^2^ with a rate of 0.58 mL/min/1.73 m^2^/year. eGFR_FAS_ was approximately stable at 117.8 mL/min/1.73 m^2^ in population younger than 40 years old, then gradually declined with a rate of 1.30 mL/min/1.73 m^2^/year in population over 40 years old. eGFR by Xiangya equation was significantly lower than those by other two equations in subjects younger than 70 years old. Similar descending trend was investigated in both males and females. The females had higher eGFR level and faster decline rate.

**Fig. 3:**
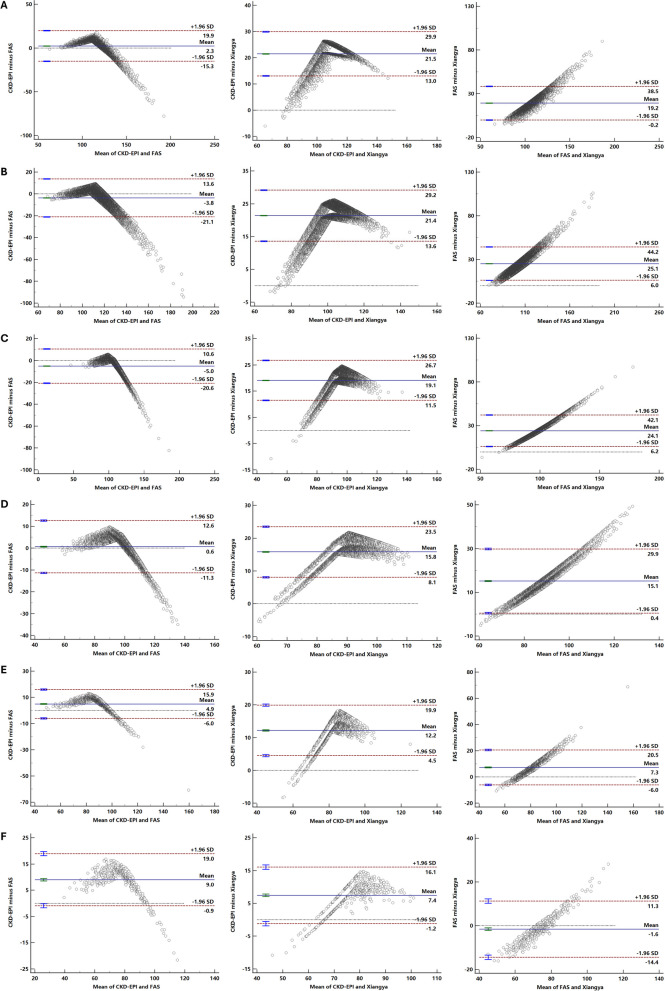
Bland–Altman plot for eGFRs by different equations comparisons according to age. Differences were plotted between eGFR by two of CKD-EPI, FAS, and Xiangya equations in participants aged **A** 18–29 years, **B** 30–39 years, **C** 40–49 years, **D** 50–59 years, **E** 60–69 years, and **F** ≥ 70 years. The blue solid line represents the mean of the differences. The red dashed line indicates 1.96 standard deviations around the mean differences. *CKD-EPI*: chronic kidney disease epidemiology collaboration equation based on serum creatinine; *FAS:* full age spectrum based on serum creatinine and age

In individuals younger than 70 years old, agreement between eGFR by CKD-EPI and FAS was good (ICC = 0.894–0.940 in males and 0.783–0.893 in females), with a mean difference of -4.0 ~ 4.7 in males and -6.0 ~ 5.4 in females. However, agreement analysis between Xiangya and other two equation showed lower ICC value (ICC = 0.388–0.786 in males and 0.282–0.732 in females) and greater mean difference of 6.5 ~ 20.9 in males and 8.6 ~ 28.2 in females, and eGFR by CKD-EPI and FAS were higher than that by Xiangya equation in most of subjects younger than 70 years old. In subgroups greater than or equal to 70 years old, agreement between two of three equations were good, and was best between FAS and Xiangya equation with a mean difference of -2.6 (95%LoA: -14.5 ~ 9.4) in males and 0.2 (95%LoA: -13.5 ~ 13.8) in females and an ICC value of 0.877 in males and 0.889 in females. Figure 3, Figs. S1, and S2 by Bland–Altman plot and Table 2 by ICC analysis shown agreement between two of three equations according to gender and age.

**Table 2:** Agreement analysis between equations by ICC according to age and gender

Age (years)	CKD-EPI ~ FAS		CKD-EPI ~ Xiangya		FAS ~ Xiangya	
	Males	Females	Males	Females	Males	Females
18–29	0.910	0.830	0.413	0.323	0.577	0.478
30–39	0.926	0.783	0.388	0.282	0.407	0.334
40–49	0.898	0.786	0.403	0.296	0.394	0.338
50–59	0.940	0.893	0.462	0.381	0.552	0.498
60–69	0.894	0.872	0.604	0.506	0.786	0.732
≥ 70	0.825	0.805	0.811	0.713	0.877	0.889

*ICC* Intraclass correlation coefficient, *CKD-EPI* Chronic kidney disease epidemiology collaboration equation based on serum creatinine, *FAS* Full age spectrum based on serum creatinine and age

Bland–Altman plot between FAS and other equations showed poor agreement when mean eGFR by above equations was large. Therefore, we plotted a scatter plot of the difference between any two equations as SCr increases. As to the comparison of FAS equation, the difference was larger in subjects with lower SCr level, which indicated that the stability of FAS equation may be poor, the distribution was more discrete. Therefore, we plotted scatter diagrams to directly reflect the difference between these equations on different SCr levels (Supplementary Fig. 3).

Reference ranges of eGFR by CKD-EPI, FAS, and Xiangya equations were shown in Table 3 according to age and gender. The reference ranges by CKD-EPI and Xiangya were narrower than which by FAS equation.

**Table 3:** Reference range of eGFR by CKD-EPI, FAS, and Xiangya equations according to age and gender

Age (years)	eGFR_CKD-EPI_		eGFR_FAS_		eGFR_Xiangya_	
	Males	Females	Males	Females	Males	Females
18–29	95.28–135.22	106.08–140.30	84.42–137.05	87.60–156.92	83.61–109.75	84.25–115.70
30–39	90.79–128.51	101.18–132.21	85.03–137.28	89.97–153.69	78.82–103.32	79.66–107.55
40–49	85.20–120.55	92.81–123.16	79.95–133.77	82.61–145.38	74.24–97.54	74.17–99.83
50–59	79.12–113.10	82.65–115.92	71.17–119.38	70.83–126.96	70.63–92.91	69.14–93.98
60–69	72.35–107.09	73.42–108.97	60.33–109.81	59.91–111.73	66.53–90.69	64.80–89.64
≥ 70	61.39–99.86	63.24–102.68	47.52–95.95	46.58–100.86	61.68–86.92	59.68–87.38

*eGFR* estimated glomerular filtration rate, *CKD-EPI* Chronic kidney disease epidemiology collaboration equation based on serum creatinine, *FAS* Full age spectrum based on serum creatinine and age

## Discussion

eGFRs by CKD-EPI, FAS or Xiangya equations were all decreased with aging. Compared with males, eGFR level was higher after adulthood but decreased faster with aging in females. Most of previous studies revealed consistent results [15, 16]. On the contrary, another study described a slower fall in GFR with age in females [17].

The declining tendency of eGFR with aging based on different equations was diverse. eGFR gradually declined with a rate of 0.82 mL/min/1.73 m^2^/year using CKD-EPI equation and 0.58 mL/min/1.73 m^2^/year using Xiangya equation; Meanwhile, eGFR_FAS_ was stable in population aged 18–39 years old and decreased with a rate of 1.30 mL/min/1.73 m^2^/year age 40 years and over. The variety tendency of eGFR_FAS_ was like that of measured GFR in previous studies. A study, including 301 Chinese healthy subjects (130 males and 171 females), revealed that mGFR of adults age under 50 years were approximately stable, and then declined with a rate of 12.2 mL/min/1.73 m^2^/decade with age [18]. A meta-analysis by Pottel et al. also revealed the construction of 40 years old as the descent inflection point of declining GFR with aging [19].

The eGFR_CKD-EPI_ level was relatively higher. A recent study demonstrated that eGFR levels by CKD-EPI formula were higher than mGFR levels in healthy European population age 50 years and above [20]. Besides, our previous studies also investigated that the GFR was overestimated using CKD-EPI equation in the older adults [12]. Meanwhile, the eGFR level by Xiangya equation was lower, especially in the younger adults, and which was also lower than measured GFR of healthy subjects in previous studies. Previous studies showed the node in the trend of GFR with age, rather than a linear trend in the development data of Xiangya equation [8]. These inconsistent trends of GFR with age may influence the accuracy of Xiangya equation and result in the quite differences between Xiangya and other two equations in healthy people. Besides, though the Xiangya equation was developed by Han Chinese, vast territory, different eating habits and body shapes may be the reason why this equation differs greatly from other equations in this study. Therefore, the establishment of an equation that is broadly applicable to Chinese, may not only consider age and sex factors excluding kidney function indicators. Thus, the application of these equations was needed to be further studied when used to estimate eGFR level.

We also observed a gender difference in the rate of eGFR decline with age, and the rate of eGFR decline with age in women is significantly faster than that in men. Differences in reserve kidney function may be an important factor in the sex difference. Previous studies have shown that renal function is preserved through renal reserve, which gradually decreases with aging [21]. Men may have higher renal function reserves to compensate for the decline in GFR due to nephron loss with age. Healthy men have been shown to be more capable than women of maintaining GFR by increasing glomerular filtration fraction [22]. In addition, kidney function is ultrafiltration to accommodate the rapidly growing metabolic demands during pregnancy [23], and a prolonged hyperfiltration state may lead to a faster subsequent decline in renal function in women [24].

We observed good to excellent agreement between CKD-EPI and FAS equations in agreement analysis among subgroups stratified by age. However, we investigated a remarkable difference between eGFR by these two equations in some of the subjects with low SCr levels. These individuals were mainly females with normal BMI, nutritive index levels but SCr lower than 0.5 mg/dL. This phenomenon may be due to the Q_SCr_ value, which was regarded as the mean or median of SCr corresponding to age or gender of the healthy population. Meanwhile, we adopted a standard of 0.9 mg/dL in males and 0.7 mg/dL in females as Q_SCr_ value according to FAS formula established for the Caucasian population [6] in this study, which may be unsuitable for the Chinese, especially younger adults with low SCr level who may have less muscle content. This may influence the accuracy in the evaluation of eGFR by FAS equation. FAS formula may not suit for evaluating eGFR in the healthy population with a low SCr level. Accuracy of estimation may be improved if multiple biomarkers are used, which may adjust for creatinine issues in older age and subjects with muscle mass extremes. Besides, it is expected to ascertain appropriate Q_SCr_ value for the Chinese further.

The agreement between Xiangya and CKD-EPI or FAS equation was poor to moderate in population under 70 years. Furthermore, the agreement analysis of Xiangya and CKD-EPI or FAS equation performs relatively well in older adults over 70 years compared with the younger adults. Therefore, these three equations are considered interchangeable for the evaluation of eGFR in older adults. However, considering the people over 70 years account for a small proportion of the total participants, further exploration is needed in the future.

Given the large difference between eGFRs by the different equations, the choice for assessing GFR can have a great impact on the assessment of public health implications. Therefore, we calculated the reference intervals in each subgroup stratified by age using CKD-EPI, FAS, and Xiangya equations. These may be helpful when evaluating the kidney function using above equations for primary medical care.

Compared with previous studies, the advantages of this study were as follows: (1) Due to the large number of healthy subjects including in this study, the variation of eGFR with age could be a reliable reference to the clinician when evaluating age-related kidney function. Besides, the reference range in healthy individuals provide reference for assessing kidney function by these equations at all ages. (2) We adopted three equations based on the recommend according to the current guideline and the development dataset of equations. Then, we firstly analyze the difference and agreement between CKD-EPI, FAS and Xiangya equations in Chinese healthy population. However, the limitation of this study is that there is no mGFR to analyze the variety tendency of GFR with increasing age and evaluate the accuracy of these equations. Considering the gold standard of renal function evaluation is too complicated and expensive to be widely used in healthy individuals, so we adopt estimated equations to calculate eGFR in this large sample population. Another limitation is that this research is a retrospective cross-sectional study.

## Conclusions

In the healthy Chinese, there are differences in evaluating eGFR and age-related decline rate of it by CKD-EPI, FAS and Xiangya equations. The clinician should pay attention to these equations- or age-related differences, especially in the older adults. Meanwhile, the difference between males and females needs attention. More verification is needed, especially in the healthy Chinese with low SCr level.

### Supplementary Information

**Additional file 1: Figure S1.** Bland-Altman plot for eGFRs by different equations comparisons according to age in the males. Differences were plotted between eGFR by two of CKD-EPI, FAS, and Xiangya equations in male participants aged (A) 18-29 years, (B) 30-39 years, (C) 40-49 years, (D) 50-59 years, (E) 60-69 years, and (F) ≥70 years. The blue solid line represents the mean of the differences. The red dashed line indicates 1.96 standard deviations around the mean differences. *CKD-EPI*: chronic kidney disease epidemiology collaboration equation based on serum creatinine; *FAS:* full age spectrum based on serum creatinine and age. **Figure S2.** Bland-Altman plot for eGFRs by different equations comparisons according to age in the females. Differences were plotted between eGFR by two of CKD-EPI, FAS, and Xiangya equations in female participants aged (A) 18-29 years, (B) 30-39 years, (C) 40-49 years, (D) 50-59 years, (E) 60-69 years, and (F) ≥70 years. The blue solid line represents the mean of the differences. The red dashed line indicates 1.96 standard deviations around the mean differences. *CKD-EPI*: chronic kidney disease epidemiology collaboration equation based on serum creatinine; *FAS:* full age spectrum based on serum creatinine and age. **Figure S3.** Scatter plot of difference between two of CKD-EPI, FAS, and Xiangya equations with increasing serum creatinine. Difference between eGFR by different equations of each individual is shown as black plot in total population, dark blue plot in the males, and orange plot in the females. The fitting line is shown as dark blue solid line in the males and orange solid line in the females. *eGFR*: estimated glomerular filtration rate; *CKD-EPI*: chronic kidney disease epidemiology collaboration equation based on serum creatinine; *FAS:* full age spectrum based on serum creatinine and age.

**Additional file 2: Table S1. **Baseline characteristics of the healthy males and females.

**Additional file 3: Table S2. **Age- and sex-specific aging trend of eGFR using three equations.

## Data Availability

Data used during the current study are available from the corresponding author upon reasonable request.

## Abbreviations

GFR: Glomerular filtration rate
mGFR: Measured glomerular filtration rate
CKD-EPI: Chronic kidney disease epidemiology collaboration
KDIGO: Kidney Disease Improving Global Outcomes
FAS: Full age spectrum
eGFR: Estimated glomerular filtration rate
BMI: Body mass index
BP: Blood pressure
FBG: Fasting blood glucose
SCr: Serum creatinine
UA: Uric acid
ALT: Alanine aminotransferase
LDL-C: Low-density lipoprotein cholesterol
HDL-C: High density lipoprotein cholesterol
TC: Total cholesterol
SD: Standard deviation
ICC: Intraclass correlation coefficient
