# Supplementary material for: Different equations for estimating age-related changes of glomerular filtration rate in the healthy population
Source: BMC Nephrol. 2023 Nov 17;24:342. doi: 10.1186/s12882-023-03397-7 (PMC10657123; doi:10.1186/s12882-023-03397-7)
Supplement: Supplementary file 1 — Additional file 1: Figure S1. Bland-Altman plot for eGFRs by different equations comparisons according to age in the males. Differences were plotted between eGFR by two of CKD-EPI, FAS, and Xiangya equations in male participants aged (A) 18-29 years, (B) 30-39 years, (C) 40-49 years, (D) 50-59 years, (E) 60-69 years, and (F) ≥70 years. The blue solid line represents the mean of the differences. The red dashed line indicates 1.96 standard deviations around the mean differences. CKD-EPI: chronic kidney disease epidemiology collaboration equation based on serum creatinine; FAS: full age spectrum based on serum creatinine and age. Figure S2. Bland-Altman plot for eGFRs by different equations comparisons according to age in the females. Differences were plotted between eGFR by two of CKD-EPI, FAS, and Xiangya equations in female participants aged (A) 18-29 years, (B) 30-39 years, (C) 40-49 years, (D) 50-59 years, (E) 60-69 years, and (F) ≥70 years. The blue solid line represents the mean of the differences. The red dashed line indicates 1.96 standard deviations around the mean differences. CKD-EPI: chronic kidney disease epidemiology collaboration equation based on serum creatinine; FAS: full age spectrum based on serum creatinine and age. Figure S3. Scatter plot of difference between two of CKD-EPI, FAS, and Xiangya equations with increasing serum creatinine. Difference between eGFR by different equations of each individual is shown as black plot in total population, dark blue plot in the males, and orange plot in the females. The fitting line is shown as dark blue solid line in the males and orange solid line in the females. eGFR: estimated glomerular filtration rate; CKD-EPI: chronic kidney disease epidemiology collaboration equation based on serum creatinine; FAS: full age spectrum based on serum creatinine and age. [file 12882_2023_3397_MOESM1_ESM.zip › Supplemental Figure legend.docx]

**Supplemental Figure Legends**

**Figure S1. Bland-Altman plot for eGFRs by different equations comparisons according to age in the males**.

Differences were plotted between eGFR by two of CKD-EPI, FAS, and Xiangya equations in male participants aged (A) 18-29 years, (B) 30-39 years, (C) 40-49 years, (D) 50-59 years, (E) 60-69 years, and (F) ≥70 years. The blue solid line represents the mean of the differences. The red dashed line indicates 1.96 standard deviations around the mean differences. *CKD-EPI*: chronic kidney disease epidemiology collaboration equation based on serum creatinine; *FAS:* full age spectrum based on serum creatinine and age.

**Figure S2. Bland-Altman plot for eGFRs by different equations comparisons according to age in the females**

Differences were plotted between eGFR by two of CKD-EPI, FAS, and Xiangya equations in female participants aged (A) 18-29 years, (B) 30-39 years, (C) 40-49 years, (D) 50-59 years, (E) 60-69 years, and (F) ≥70 years. The blue solid line represents the mean of the differences. The red dashed line indicates 1.96 standard deviations around the mean differences. *CKD-EPI*: chronic kidney disease epidemiology collaboration equation based on serum creatinine; *FAS:* full age spectrum based on serum creatinine and age.

**Figure S3. Scatter plot of difference between two of CKD-EPI, FAS, and Xiangya equations with increasing serum creatinine.**

Difference between eGFR by different equations of each individual is shown as black plot in total population, dark blue plot in the males, and orange plot in the females. The fitting line is shown as dark blue solid line in the males and orange solid line in the females. *eGFR*: estimated glomerular filtration rate; *CKD-EPI*: chronic kidney disease epidemiology collaboration equation based on serum creatinine; *FAS:* full age spectrum based on serum creatinine and age.
