# Supplementary material for: Different equations for estimating age-related changes of glomerular filtration rate in the healthy population
Source: BMC Nephrol. 2023 Nov 17;24:342. doi: 10.1186/s12882-023-03397-7 (PMC10657123; doi:10.1186/s12882-023-03397-7)
Supplement: Supplementary file 2 — Additional file 2: Table S1. Baseline characteristics of the healthy males and females. [file 12882_2023_3397_MOESM2_ESM.docx]

**Table S1 Baseline characteristics of the healthy males and females.**

| Variable | All subjects | | 18-29years | | 30-39years | | 40-49years | | 50-59years | | 60-69years | | ≥70years | |
| --- | --- | --- | --- | --- | --- | --- | --- | --- | --- | --- | --- | --- | --- | --- |
|  | Males  (n=16096) | Females  (n=20817) | Males  (n=2955) | Females  (n=4895) | Males  (n=5474) | Females  (n=8904) | Males  (n=3930) | Females  (n=4398) | Males  (n=2480) | Females  (n=1862) | Males  (n=943) | Females  (n=582) | Males  (n=314) | Females  (n=176) |
| SBP (mmHg) | 120.56(10.29) | 113.89(11.20)^*^ | 120.97(9.78) | 113.05(10.63)^*^ | 119.44(10.11) | 112.13(11.60)^*^ | 119.72(10.39) | 114.66(11.45)^*^ | 121.53(10.36) | 118.55(11.31)^*^ | 124.17(10.52) | 123.24(11.19) | 128.39(8.96) | 126.37(10.22)^*^ |
| DBP (mmHg) | 74.35(7.59) | 69.81(8.06)^*^ | 72.63(7.55) | 69.66(7.79)^*^ | 73.50(7.49) | 69.37(7.86)^*^ | 75.33(7.44) | 70.00(8.38)^*^ | 76.38(7.42) | 71.39(8.47)^*^ | 75.93(7.16) | 71.61(8.33)^*^ | 72.19(7.49) | 68.80(8.40)^*^ |
| BMI (kg/m^2^) | 23.74(2.31) | 21.96(2.15)^*^ | 23.27(2.39) | 21.37(1.98)^*^ | 23.70(2.32) | 21.88(2.13)^*^ | 23.96(2.25) | 22.33(2.11)^*^ | 23.99(2.17) | 22.55(2.13)^*^ | 23.90(2.28) | 22.98(2.30)^*^ | 23.71(2.34) | 23.43(2.63) |
| HDL (mmol/L) | 1.33(0.23) | 1.52(0.27)^*^ | 1.31(0.20) | 1.52(0.25)^*^ | 1.31(0.22) | 1.51(0.26) | 1.33(0.23) | 1.54(0.28)^*^ | 1.34(0.24) | 1.56(0.28)^*^ | 1.37(0.25) | 1.52(0.28)^*^ | 1.37(0.26) | 1.50(0.29)^*^ |
| LDL (mmol/L) | 3.02(0.53) | 2.89(0.52)^*^ | 2.91(0.53) | 2.77(0.51)^*^ | 3.01(0.52) | 2.85(0.51)^*^ | 3.09(0.51) | 2.98(0.50)^*^ | 3.11(0.52) | 3.15(0.47)^*^ | 3.03(0.56) | 3.13(0.52)^*^ | 2.84(0.67) | 2.97(0.57)^*^ |
| TG (mmol/L) | 1.21(0.42) | 0.97(0.37)^*^ | 1.11(0.40) | 0.88(0.32)^*^ | 1.21(0.42) | 0.95(0.35)^*^ | 1.26(0.43) | 1.01(0.36)^*^ | 1.24(0.41) | 1.15(0.40)^*^ | 1.24(0.43) | 1.31(0.41)^*^ | 1.15(0.38) | 1.28(0.39)^*^ |
| TC (mmol/L) | 4.91(0.69) | 4.86(0.70)^*^ | 4.73(0.70) | 4.67(0.70)^*^ | 4.88(0.68) | 4.78(0.69)^*^ | 5.00(0.66) | 5.00(0.66) | 5.02(0.66) | 5.23(0.60)^*^ | 4.98(0.71) | 5.23(0.68)^*^ | 4.73(0.86) | 5.04(0.73)^*^ |
| FBG (mmol/L) | 5.15(0.48) | 5.02(0.43)^*^ | 5.00(0.41) | 4.91(0.40)^*^ | 5.06(0.41) | 4.99(0.40)^*^ | 5.19(0.46) | 5.06(0.42)^*^ | 5.31(0.52) | 5.18(0.48)^*^ | 5.44(0.55) | 5.31(0.49)^*^ | 5.59(0.59) | 5.48(0.60) |
| ALB (g/L) | 46.18(2.22) | 44.85(2.14)^*^ | 47.55(1.77) | 45.69(2.10)^*^ | 46.8(1.88) | 45.03(2.00)^*^ | 45.75(2.04) | 44.07(2.04)^*^ | 44.99(2.05) | 44.14(2.08)^*^ | 44.27(2.03) | 43.81(1.90)^*^ | 42.93(2.37) | 42.75(2.06) |
| UA (umol/L) | 341.72(46.82) | 265.41(50.73)^*^ | 349.32(44.22) | 270.50(51.50)^*^ | 344.08(45.76) | 263.15(50.07)^*^ | 338.30(47.46) | 257.65(48.91)^*^ | 337.25(47.40) | 274.79(50.60)^*^ | 334.10(50.60) | 279.40(52.43)^*^ | 330.29(51.27) | 286.72(58.72)^*^ |
| BUN (mmol/L) | 5.13(1.10) | 4.46(1.05)^*^ | 4.97(1.06) | 4.27(1.01)^*^ | 5.05(1.07) | 4.39(1.00)^*^ | 5.15(1.09) | 4.51(1.04)^*^ | 5.33(1.14) | 4.92(1.08)^*^ | 5.33(1.14) | 5.13(1.13)^*^ | 5.49(1.32) | 5.26(1.24) |
| Scr (mg/dL) | 0.88(0.11) | 0.63(0.09)^*^ | 0.88(0.11) | 0.63(0.09)^*^ | 0.88(0.10) | 0.63(0.08)^*^ | 0.87(0.11) | 0.64(0.08)^*^ | 0.87(0.11) | 0.66(0.09)^*^ | 0.87(0.12) | 0.67(0.10)^*^ | 0.89(0.13) | 0.69(0.12)^*^ |

Values were presented as mean (standard deviation).

Abbreviations: *SBP* systolic blood pressure; *DBP* diastolic blood pressure; *BMI* body mass index; *HDL-C* high density lipoprotein cholesterol; *LDL-C* low-density lipoprotein cholesterol; *TG* triglyceride; *TC* total cholesterol; *FBG*: fasting blood glucose; *ALB* albumin; *UA* uric acid; *BUN* blood urea nitrogen.

*P<0.05, compared with males.
