# Supplementary material for: Different equations for estimating age-related changes of glomerular filtration rate in the healthy population
Source: BMC Nephrol. 2023 Nov 17;24:342. doi: 10.1186/s12882-023-03397-7 (PMC10657123; doi:10.1186/s12882-023-03397-7)
Supplement: Supplementary file 3 — Additional file 3: Table S2. Age- and sex-specific aging trend of eGFR using three equations. [file 12882_2023_3397_MOESM3_ESM.docx]

**Table S2 Age- and sex-specific aging trend of eGFR using three equations.**

| Age  (year) | All | Male | Female |
| --- | --- | --- | --- |
|  | **eGFR_CKD-EPI_** | | |
| 18-29 | 120.20(119.98-120.42) | 115.25(114.88-115.62) | 123.19(122.94-123.43) |
| 30-39 | 114.01(113.86-114.16) | 109.65(109.39-109.90) | 116.70(116.53-116.86) |
| 40-49 | 105.57(105.39-105.76) | 102.88(102.59-103.16) | 107.99(107.76-108.22) |
| 50-59 | 97.47(97.21-97.73) | 96.11(95.77-96.45) | 99.29(98.90-99.67) |
| 60-69 | 90.28(89.83-90.73) | 89.72(89.15-90.29) | 91.20(90.46-91.93) |
| ≥70 | 81.46(80.58-82.35) | 80.63(79.54-81.72) | 82.96(81.46-84.46) |
|  | **eGFR_FAS_** | | |
| 18-29 | 117.92(117.54-118.30) | 110.74(110.25-111.22) | 122.26(121.76-122.75) |
| 30-39 | 117.77(117.51-118.03) | 111.16(110.80-111.51) | 121.83(121.49-122.17) |
| 40-49 | 110.63(110.30-110.96) | 106.86(106.43-107.29) | 113.99(113.52-114.47) |
| 50-59 | 96.83(96.43-97.22) | 95.28(94.79-95.76) | 98.90(98.25-99.55) |
| 60-69 | 85.36(84.71-86.00) | 85.07(84.26-85.87) | 85.82(84.74-86.90) |
| ≥70 | 72.45(71.30-73.60) | 71.74(70.36-73.11) | 73.72(71.66-75.78) |
|  | **eGFR_Xiangya_** | | |
| 18-29 | 98.73(98.56-98.90) | 96.68(96.44-96.92) | 99.97(99.75-100.20) |
| 30-39 | 92.64(92.53-92.75) | 91.07(90.90-91.23) | 93.60(93.46-93.75) |
| 40-49 | 86.48(86.34-86.61) | 85.89(85.71-86.08) | 87.00(86.81-87.19) |
| 50-59 | 81.68(81.50-81.86) | 81.77(81.55-81.99) | 81.56(81.28-81.85) |
| 60-69 | 78.08(77.76-78.39) | 78.61(78.21-79.00) | 77.22(76.70-77.74) |
| ≥70 | 74.02(73.43-74.61) | 74.30(73.58-75.01) | 73.53(72.48-74.58) |

Values were presented as mean (confidence interval).
